# The developmental environment modulates mating‐induced aggression and fighting success in adult female *Drosophila*

**DOI:** 10.1111/1365-2435.13214

**Published:** 2018-09-28

**Authors:** Eleanor Bath, Juliano Morimoto, Stuart Wigby

**Affiliations:** ^1^ Department of Zoology, Edward Grey Institute University of Oxford Oxford UK; ^2^ Christ Church College University of Oxford Oxford UK; ^3^ Department of Biological Sciences Macquarie University North Ryde NSW Australia; ^4^ Programa de Pós‐Graduação em Ecologia e Conservação Federal University of Paraná Curitiba Brazil

**Keywords:** *Drosophila*, early‐life effects, female–female competition, resources, seminal fluid, sex peptide, sexual selection, social selection

## Abstract

1. Competition over access to resources early in life can influence development, and, in turn, affect competitive phenotypes in reproductive adults. Theory predicts that competition between adult females should be especially context‐dependent, because of constraints imposed by high costs of reproduction. However, the potential impact of developmental environments on competition in adult females remains little understood.
2. In *Drosophila melanogaster*, the developmental environment can strongly influence adult condition, and prime adult competitive behaviour. In this species, female–female aggression is dependent on reproductive state and increases after mating due to the receipt of sperm and seminal fluid components. However, the effects of the developmental environment on adult female aggression, and any potential interactions with mating status, are unknown.
3. To address this problem, we first raised flies at low and high larval density, which altered competition over limited resources, produced large and small adult females, respectively, and potentially primed them for differing levels of adult competition. We then fought the resulting adult females, either as virgins, or after receiving aggression‐stimulating ejaculates at mating, to test for interacting effects.
4. We found, as expected, that mating elevated contest duration. However, this mating‐induced boost in aggression was strongly exacerbated for high density (small) females. Low density (large) females won more contests overall, but were not more successful in fights after mating. In contrast, mating increased the fighting success in females raised in high density environments.
5. Our results suggest that individuals who experience competitive, resource‐limited, rearing conditions are more sensitive to the aggression‐stimulating effects of the male ejaculate. This finding highlights the importance of the developmental environment in mediating adult social interactions and provides support for the theory that female–female aggression should be highly context‐dependent.

A http://onlinelibrary.wiley.com/doi/10.1111/1365-2435.13214/suppinfo is available for this article.

## 1 INTRODUCTION

Developmental environments can vary in many ways, including nutrient availability and population density (Boggs, 1981). Variation in the developmental environment between individuals can lead to long‐lasting differences in fitness traits, such as body size, attractiveness, development rate and readiness to face competition (Bonduriansky, 2001; Gage, 1995; Kasumovic & Brooks, 2011; Katsuki, Toquenaga, & Miyatake, 2013; Lüpold, Manier, Ala‐Honkola, Belote, & Pitnick, 2011). In holometabolous insects, for example, adult body size is fixed at eclosion and determined primarily by the amount of food consumed during the larval stage (Boggs, 1981; Clancy & Kennington, 2001). Larger males are often more successful in intrasexual contests and have higher mating and reproductive success than smaller males in a variety of species (Cowlishaw & Dunbar, 1991; Kelly, 2008; McCann, 1981; McGraw et al., 2007; Moczek, 1998; Morimoto, Pizzari, & Wigby, 2016). Resource availability during development can also influence how individuals value resources as adults, influencing their motivation and ability to compete over access to food or other resources (Hopwood, Moore, & Royle, 2014; Royle, Lindström, & Metcalfe, 2005; Wigby, Perry, Kim, & Sirot, 2016). Variation amongst individuals in their motivation to compete may have implications for contest dynamics, with changes in motivation enabling smaller individuals to overcome size differences and improve their success in competitive scenarios—for example, males of the cichlid, *Herichthys cyanoguttatum* (Draud, Macías‐Ordóñez, Verga, & Itzkowitz, 2004) and the hummingbird, *Archilochus alexandri* (Ewald, 1985). Additionally, population density during development can act as an indicator of future competition, so individuals can alter their investment in competitive traits in response to their developmental environment (Cotton, Fowler, & Pomiankowski, 2004; Gage, 1995; He & Miyata, 1997; Katsuki et al., 2013; Sentinella, Crean, & Bonduriansky, 2013; Wigby et al., 2016). Furthermore, phenotypic variation between individuals generated as a result of varying developmental environments can influence the growth and survival of groups and populations (Morimoto, Ponton, Tychsen, Cassar, & Wigby, 2017) and determine the operation of key evolutionary processes such as sexual selection and sexual conflict (Morimoto et al., 2016).

Past research on how the developmental environment can influence contest dynamics has largely focused on competition amongst males (Amitin & Pitnick, 2007; Edward & Chapman, 2012; Gage, 1995; He & Miyata, 1997; Pitnick, 1991; Pitnick & Garcia‐Gonzalez, 2002). However, the developmental environment and adult competition over resources are also potentially important mediators of fitness in females (Clutton‐Brock & Huchard, 2013; Stockley & Campbell, 2013). Moreover, female–female competition has been suggested to be more sensitive to environmental variation than male–male competition, due to the higher costs and lower benefits faced by females from engaging in aggressive behaviours (Clutton‐Brock, 2009; Stockley & Campbell, 2013). Despite this, little work has investigated the potential role of the developmental environment in influencing female–female competition and levels of aggression (though see Cain & Ketterson, 2013; Cain & Langmore, 2016 for two observational field studies in birds). Hormones experienced during development have been shown to influence levels of both juvenile and adult aggression in a variety of bird species (Bentz, Becker, & Navara, 2016; Cordero, Ansermet, & Sandi, 2013; Müller, Dijkstra, & Groothuis, 2009). One study in the spotted hyena (*Crocuta crocuta*) found that females exposed to higher levels of androgens during gestation displayed higher levels of aggression as cubs (Dloniak, French, & Holekamp, 2006), which acts as a good indicator of success in adult competition to acquire social rank, leading to higher reproductive success (Holekamp & Smale, 1993). These results show an association between the developmental environment and female fitness—by altering individuals’ chances of success in juvenile and adult competition—but few studies have investigated these effects outside of birds and mammals.

The developmental environment has dramatic effects on aspects of female fitness in insects, for example, with larger females being more fecund and more attractive to males (Bonduriansky, 2001). These differences in female attractiveness result in flow‐on effects on reproductive success in terms of size of ejaculate transfer, rates of male harassment and amount of paternal investment in offspring (Harley et al., 2013; Long, Pischedda, Stewart, & Rice, 2009; Mahr, Griggio, Granatiero, & Hoi, 2012; Wigby et al., 2016), but the effects on female–female competition are poorly understood. To address this problem, we investigated whether the developmental environment influences contest outcome and dynamics in female fruit flies, *Drosophila melanogaster*. Previous work has shown that male *D. melanogaster* raised at low larval density—which eclose larger than those raised at high larval density—are more successful in intra‐sexual competition (Bangham, Chapman, & Partridge, 2002; Lefranc & Bundgaard, 2000; Miller & Thomas, 1958; Pitnick & Garcia‐Gonzalez, 2002), but whether the same pattern holds for females is unknown. We previously demonstrated that ejaculate components transferred at mating increase the duration of female aggression in female *D. melanogaster* but detected no effect of mating on fighting success (Bath et al., 2017). Crucially, our previous work was conducted on females all raised in benign, resource‐rich and low density developmental environments. The developmental environment can influence the quantity of ejaculate received by females (Lüpold et al., 2011) as well as how females respond to it: low density (large females) receive absolutely more of the seminal protein “sex peptide,” but respond less strongly to its receptivity‐inhibiting effects than high‐density (small) females, perhaps because small females receive a larger dose relative to body size (Wigby et al., 2016). This raises the possibility that the developmental environment could also influence how female aggression responds to mating. To test these ideas, we manipulated larval density during development and measured the effects on mating‐induced female aggression in adulthood. We had 2 main predictions:

1. Females raised at low larval density would win more fights against females raised at high larval density due to the competitive advantage of increased body size, but that, based on past findings (Bath et al., 2017), mating would not influence contest outcome.
2. EITHER, females raised at low larval density (who eclose larger) will show a greater increase in mating‐induced aggression, because they receive an absolutely larger ejaculate (Lüpold et al., 2011; Wigby et al., 2016), OR females raised at high larval density (who eclose smaller) will show a greater increase in mating‐induced aggression, because of the larger ejaculate‐to‐body size ratio (Wigby et al., 2016).

## 2 MATERIALS AND METHODS

### 2.1 Fly stocks and culture

We used the Dahomey wild‐type stock, which was first collected in Benin, Africa, in 1970. Flies have been maintained since then in large, outbred population cages with overlapping generations (Bath et al., 2017; Partridge & Farquhar, 1983). Fly culture and experiments were conducted at 25°C on a 12:12 light: dark cycle in a non‐humidified room. Adult flies were kept on standard fly medium (Lewis, 1960), with no access to live yeast except where stated.

### 2.2 Larval diet manipulation

Larval density was manipulated to generate females of different adult body size. Larval density and nutrition affect adult body size in *D. melanogaster* (Clancy & Kennington, 2001), which has subsequent effects on female fecundity and attractiveness (Long et al., 2009; Lüpold et al., 2011; Wigby et al., 2016), as well as male success in sexual competition (Bangham et al., 2002; Dow & von Schilcher, 1975; Lefranc & Bundgaard, 2000; Miller & Thomas, 1958; Partridge & Farquhar, 1983; Partridge, Hoffman, & Jones, 1987; Pitnick & Garcia‐Gonzalez, 2002). Manipulating larval density is a widely used technique which changes the amount of food available to each larva while keeping the starting food composition constant (Lupold et al., 2016; Lüpold et al., 2011; McGraw et al., 2007; Morimoto et al., 2016, 2017 ; Pitnick, 1991; Sørensen & Loeschcke, 2001; Wigby et al., 2016).

We manipulated egg density following methods described in Clancy and Kennington (2001. We had two treatments: high density = ~70 larvae per ml of standard fly food (~420 larvae in a 34‐ml plastic vial containing 6 ml of food), and low density = ~5 larvae per ml of food (~225 larvae in a 75‐ml plastic bottle containing ~45 ml of food). To check that our larval density manipulations worked to alter adult body size, we froze a subset of females directly after recording their behaviour. We then defrosted and weighed a subset of females (minimum of 55 females from each larval density vs. mating treatment).

### 2.3 Experimental design

Flies were collected within 7 hr of eclosion using ice anaesthesia to ensure virginity. Females were then kept in individual vials with standard fly medium (Lewis, 1960), but no live yeast. Males were kept in groups of 15–20 individuals in vials with ad libitum live yeast. We used virgin males from the low density treatment as mates for all females in “mated” treatments.

Three days post‐eclosion, virgin females were marked with acrylic paint (red or yellow) on the thorax to allow individual identification (Nilsen, Chan, Huber, & Kravitz, 2004), and returned to individual vials. After 24 hr, females allocated to the “mated” treatments were placed individually with one male and the pair was observed until a single mating occurred. Females that did not mate within 5 hr after the onset of the mating trials were discarded. After each female mated exactly once, males were immediately discarded and all females (i.e., both mated and virgins) were individually transferred to fresh vials with standard fly media and no live yeast. We kept females in these vials overnight and froze the vials after the female was removed each day. We subsequently counted eggs in each vial to determine differences in egg production due to mating and larval density treatment.

The following morning (5 days post‐eclosion), females were individually placed in vials containing damp cotton wool and no food for 2 hr (Edwards, Rollmann, Morgan, & Mackay, 2006), after which pairs of females were simultaneously aspirated from these vials into the contest arena. The arena was a circular plastic arena of 20 mm diameter, containing an Eppendorf tube cap (diameter 5 mm) filled with standard fly food media and a ~2 µl drop of yeast paste, providing a limited resource for females to compete over (Bradley & Simmons, 1997). All ten possible combinations of larval density and mating status for the two competitors were tested (e.g., high mated vs. high mated, high mated vs. high virgin, high mated vs. low mated, high mated vs. low virgin—sample sizes provided in figure legends). After introducing females to the contest arena, the fighting pair was allowed to acclimatize for 5 min, as is a common procedure in *Drosophila* aggression studies (Dierick & Greenspan, 2006, 2007; Edwards et al., 2006), after which their behaviour was recorded for 30 min using a Toshiba Camileo X400 HD video camera.

### 2.4 Behavioural analysis

Videos were scored blind to treatment, although it was not possible to conceal obvious differences in size due to the larval density treatment. Fighting behaviours (head‐butt, shove, retreat (Nilsen et al., 2004)) and feeding behaviours were recorded. We recorded the number and duration of encounters, which female initiated each encounter, the outcome of each encounter (i.e., win, lose, draw) and time spent feeding, using the program JWatcher +Video (Blumenstein, Evans, & Daniels, 2006).

### 2.5 Statistical analysis

We used generalized linear models (GLMs) with a Gamma error distribution to test the effects of larval density and mating status on body mass, total feeding duration and total contest duration, as these response variables were continuous and most closely fit a Gamma distribution. As Gamma error distributions use a logistic link function, we added 1 to all scores of total contest and feeding duration prior to transformation to include replicates with scores of 0, as the Gamma error distribution cannot incorporate 0 values. We controlled for pseudoreplication in all our analyses; for analyses of contest and feeding duration, we used dyad as the unit of replication, not individual flies, as results from flies in the same dyad were not independent. For analyses of egg production (which was measured prior to contests) and body mass (after contests), we used individual as the unit of replication as these factors were not affected by females’ competitors. To analyse egg production, we used a GLM with a Poisson distribution and *quasi* extension, as our data were count data and overdispersed, which compromises the use of a strict Poisson model.

We used a GLM with a negative binomial distribution to analyse how the number of attacks initiated by females differed in relation to larval density and mating status. We controlled for pseudoreplication by only including one randomly selected individual in each dyad in our analysis.

We also calculated the “proportion of encounters won” as the number of encounters won by the focal individual divided by the total number of decided encounters (where there was a winner and loser). We tested the effects of focal larval density treatment, focal mating status, competitor larval density treatment and competitor mating status on the “proportion of encounters won” by fitting a GLM with a Binomial error distribution and *quasi* extension, which accounted for the overdispersion of the data. For this analysis, we incorporated data from all individuals, but controlled for nonindependence of the data points of individuals from the same dyad as follows: We calculated the dispersion and the degrees of freedom of the model using the number of dyads (rather than the number of individuals) and doubled the sum of the residuals from the fitted model (McCullagh & Nelder, 1989).

In contest duration, we detected one high outlier in the HVHV treatment using the Grubbs outlier test. To address this, we winsorized the data by replacing the outlier with the next most extreme value within the HVHV treatment (Quinn & Keough, 2002).

As there were multiple levels of interactions for our models examining contest duration, feeding duration, contest initiations and the proportion of encounters won, we used AIC model comparison to choose the best fitting model. Unless explicitly stated, we report here the results from the best fitting model as judged by having the lowest AIC value. We report all models within 5 AIC of the best model in the Supporting information, but for simplicity we restrict the focus of our analysis to the best model for each test. For the proportion of encounters won, we used quasi‐AIC (“q‐AIC”) model comparison as it was not possible to acquire AIC information for a GLM with a quasibinomial distribution. For the best model in each case, we used Type I sequential sum of squares ANOVA to determine the significance of all main effects and interactions, where the effects of each factor are calculated taking into account the deviance explained by previous factors (using the “ANOVA” function in the base package of R). All analyses were conducted in R version 3.0 (R Core Team, 2012). We conducted our AIC analyses using the “step” command in R, which uses forward and backwards‐fitting to generate the best model using AIC. We used the “bblme” package for R to calculate quasi‐AIC values (Bolker & R Development Core Team, 2017).

## 3 RESULTS

### 3.1 Body mass

**Figure 1:**
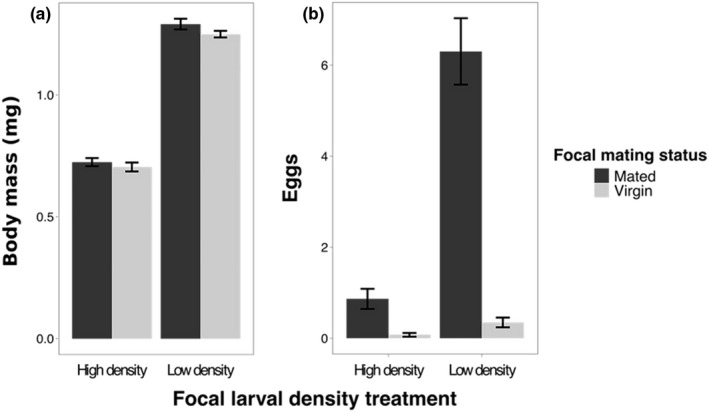
Body mass and egg production. (a) Low density females were significantly heavier than females raised at high density. Sample sizes: HM = 59, HV = 66, LM = 66, LV = 57. (b) Low density females and mated females produced more eggs over 24 hr than high density and virgin females. Sample sizes (no. of females laying eggs): HM = 110, HV = 107, LM = 107, LV = 118

As expected based on previous studies, adult females from the low density larval treatment weighed significantly more than females from the high density larval treatment (low density: 1.27 mg ± 0.01; high density: 0.72 mg ± 0.01; GLM: Dev_1, 245_ = 20.116, *p* < 0.001; Figure 1a). There was no effect of mating status on body mass (Dev_1,246_ = 0.002, *p* = 0.79), or interaction between mating status and larval density treatment (Dev_1,244_ = 0.003, *p* = 0.91).

### 3.2 Egg production

Mated females laid significantly more eggs than virgin females the day before participating in contests, regardless of larval density (Dev_1,440_ = 784.1, *p* < 0.001; Figure 1b). Females from the low density larval treatment produced significantly more eggs (low mated: 6.3 ± 0.73; low virgin: 0.35 ± 0.11) than females from the high density treatment (high mated: 0.87 ± 0.22; high virgin: 0.07 ± 0.04; Dev_1,439_ = 529.39, *p* < 0.001; Figure 1b). There was no interaction between mating status and larval density (Dev_1,438_ = 1.19, *p* = 0.64).

### 3.3 Feeding duration

**Figure 2:**
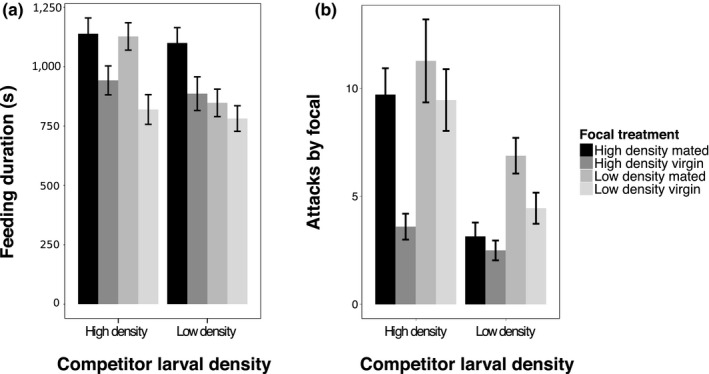
Feeding duration and attacks initiated by focal females. (a) Low density females initiate contests more than high density females, and all females attack more when their competitor is from high density. Competitor mating status is not shown as it was not significant in the model, while competitor larval density was. Sample sizes: HM vs. H: 28, HV vs. H: 18, LM vs. H: 27, LV vs. H: 35, HM vs. L: 35, HV vs. L: 28, LM vs. L: 18, LV vs. L: 33. (b) Mated females and high density females spent more time feeding, and females spent more time feeding when facing a high density competitor. Competitor mating status is not shown as it was not significant in the model, while competitor larval density was. Sample sizes: HM vs. H: 84, HV vs. H: 82, LM vs. H: 90, LV vs. H: 92, HM vs. L: 92, HV vs. L: 90, LM vs. L: 86, LV vs. L: 96

Using AIC model comparison, we found that the best model for feeding duration contained only the main factors focal mating status, focal larval density and competitor larval density (AIC = 6837.6, Deviance = 206.59). Mated females spent significantly more time feeding than virgin females (Dev_1,220_ = 4.4, *p* < 0.001, Figure 2b, Supporting information Table S1b). Females raised at high density spent more time feeding than females raised at low density (Dev_1,219_ = 2.38, *p* = 0.002), while females that faced a high density competitor spent more time feeding than females that faced a low density competitor (Dev_1,218_ = 1.23, *p* = 0.026). Several alternative models were within 5 AIC points of the best model, and these models included various two‐way interactions between all three factors (see Supporting information Table S1a).

### 3.4 Initiating contests

The best model for explaining which female initiated contests contained the factors focal mating status (Dev_1,217_ = 20.07, *p* < 0.001), focal larval density (Dev_1,218_ = 2.16, *p* < 0.001), competitor larval density (Dev_1,220_ = 23.15, *p* < 0.001, Figure 2a, Supporting information Table S2b) and the interaction between focal mating status and focal larval density (even though this interaction was not significant: Dev_1,216_ = 2.99, *p* = 0.084). Low density females initiated more attacks than high density females, as did mated females of both treatments. Females attacked more if their competitor was raised at high larval density, and there was a non‐significant trend for the effect of mating status to be more pronounced in high density females, where mated females initiated a much higher proportion of encounters than virgins. There were similar models that were within 5 AIC points of the best model fit, with additional interactions included (Supporting information Table S2a).

### 3.5 Proportion of encounters won

**Figure 3:**
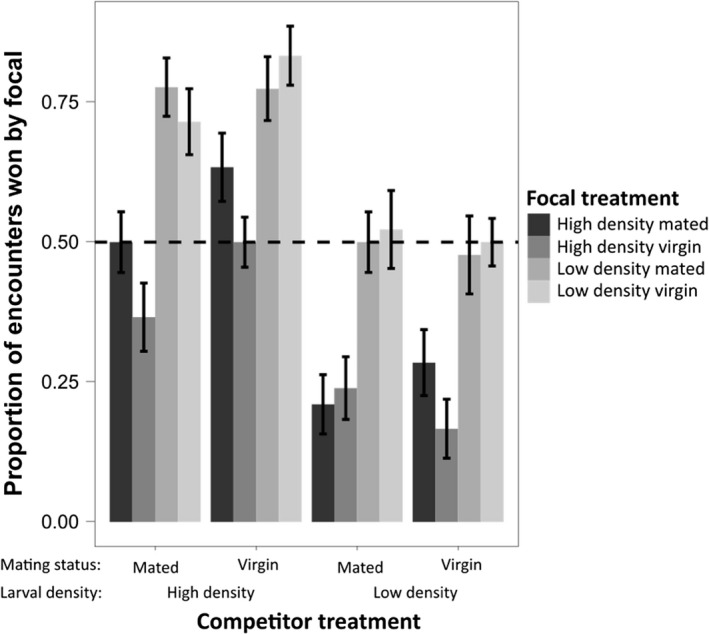
Proportion of encounters won by focal female treatment split by competitor female mating status and larval density treatment. Different shaded bars represent different focal treatments (i.e., combined mating status and larval density treatment). All combinations of focal and competitor are shown, so some treatments are the reciprocals of others; for example, high density mated focal vs. high density virgin competitor is the exact reciprocal of high density virgin focal vs. high density mated competitor. Sample sizes (no. of pairs of females): HMHM: 22, HVHM: 20, HVHV: 21, LMHM: 22, LMHV: 23, LMLM: 20, LVHM: 24, LVHV: 22, LVLM: 23, LVLV: 25

Using quasi‐AIC comparison, we identified the best model to explain the proportion of encounters won by female flies. The best model included all main effects as well as an interaction between focal mating status and focal larval density and an interaction between competitor mating status and competitor larval density (q‐AIC = 610.36, *df* = 7). In summary, females from the low density larval treatment won a higher proportion of encounters, independently of their mating status, compared to females from the high density larval treatment (Dev_1,220_ = 539.71, *p* < 0.001, Figure 3; Supporting information Table S3b), and all individuals won more when facing a high density female (Dev_1,218_ = 789.08, *p* < 0.001). There was a marginally non‐significant trend for individuals to be more successful against virgin competitors when they were from a high density treatment, rather than a low density treatment (Dev_1,215_ = 22.84, *p* = 0.074). Or conversely, mated females from the high density larval treatment won a higher proportion of encounters than virgin females from the high density treatment; that is, there was a developmental environment‐dependent effect of mating on the proportion of encounters won by females. There were no significant effects of focal mating status (Dev_1,219_ = 16.67, *p* = 0.127), competitor mating status (Dev_1,217_ = 20.42, *p* = 0.09) or the interaction between focal larval density and mating status (Dev_1,216_ = 16.86, *p* = 0.125). There were also other models within 5 q‐AIC points of the best fit, which incorporated additional interactions (Supporting information Table S3a).

### 3.6 Contest duration

Using AIC comparisons, we established that the best model fit for contest duration contained the main effects of focal mating status, competitor mating status and competitor larval density, as well as an interaction between focal mating status and competitor larval density (AIC = 1369.5, Dev = 114.66). There were multiple models within 5 AIC points of this model, including the full model with all two‐, three‐, and four‐way interactions included. Here, we focus on the best model, but full model comparisons are given in the Supplementary Material (Supporting information Table S4a–c).

As expected, mated females fought for longer than virgin females (Dev_1,220_ = 8.7, *p* < 0.001, Supporting information Table S4b) and fought for longer when fighting against mated competitors (Dev_1,219_ = 5.22, *p* = 0.001). Finally, there was a marginally non‐significant interaction between focal mating status and competitor larval density, where the difference between mated and virgin females tended to be greater when facing a high‐density competitor (Dev_1,217_ = 1.76, *p* = 0.056). There was no significant effect of competitor larval density (Dev_1,218_ = 1.24, *p* = 0.11).

## 4 DISCUSSION

We tested whether mating status and larval density influence female competition in *D. melanogaster*. Consistent with previous findings (Bath et al., 2017), contest duration was strongly influenced by mating status, whereby pairs of mated females fought for longer than mixed pairs, or pairs with two virgin females. However, we found that high density larval environments strongly exacerbated the difference in contest duration between mated and virgin female pairs. Furthermore, our data show that while contest outcomes were heavily influenced by larval density—whereby females raised at low density won a higher proportion of encounters than females raised at high density—mating status had an interacting effect. High density‐raised, mated females initiated more attacks and won a higher proportion of encounters than virgin high density females, but this effect was absent in the low density treatment, suggesting a developmental environment‐specific effect of mating on aggression.

Differential resource valuation amongst females may play a major role in determining the length and outcome of female aggressive encounters across a wide range of species (Clutton‐Brock & Huchard, 2013; Draud et al., 2004; Elias, Botero, Andrade, Mason, & Kasumovic, 2010). Changes in levels of aggression are associated with reproductive status and stage of reproduction in mammals, lizards, fish and insects suggesting that females value resources differently according to their current reproductive state. For example, pregnant females display more aggression in White's skinks (*Egernia whitii*: Sinn, While, & Wapstra, 2008), mosquitofish (*Gambusia holbrooki*: Seebacher, Ward, & Wilson, 2013) and mice (*Musculus musculus*: Palanza, Re, Mainardi, Brain, & Parmigiani, 1996), while cycling or oestrous females display higher levels of aggression in red deer (*Cervus elaphus*: Bebié & McElligott, 2006) and chacma baboons (*Papio ursinus*: Huchard & Cowlishaw, 2011).

**Figure 4:**
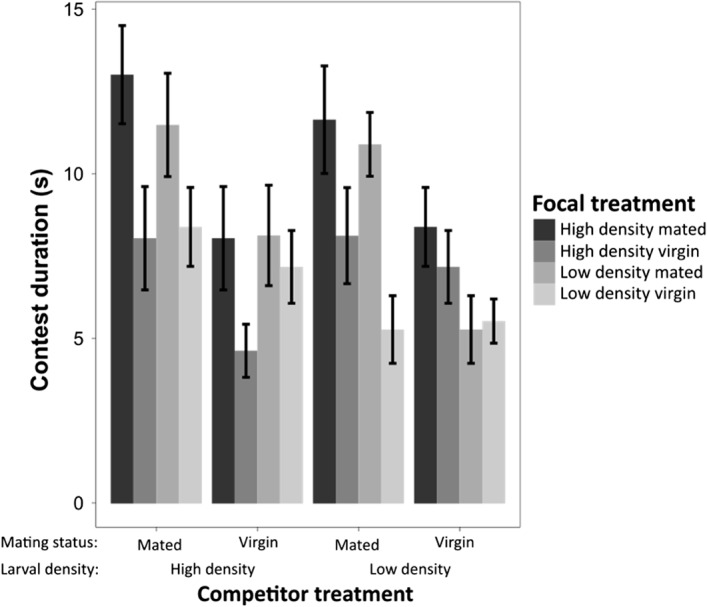
Contest duration by focal female treatment split by competitor female mating status and larval density treatment. Different shaded bars represent different focal treatments (i.e., combined mating status and larval density treatment). All combinations of focal and competitor are shown, so some treatments are the reciprocals of others; for example, high density mated focal versus high density virgin competitor is the exact reciprocal of high density virgin focal versus high density mated competitor. Sample sizes (no. of pairs of females): HMHM: 22, HVHM: 20, HVHV: 21, LMHM: 22, LMHV: 23, LMLM: 20, LVHM: 24, LVHV: 22, LVLM: 23, LVLV: 25

Our finding that mated females fought for longer than virgin females agrees with results from previous studies in this and other species, that reproductive stage can influence female contest dynamics (Bath et al., 2017; Huchard & Cowlishaw, 2011; Nilsen et al., 2004; Palanza et al., 1996; Seebacher et al., 2013). Mated female *D. melanogaster* require diets with a higher ratio of protein to carbohydrate to facilitate increased egg production (Barnes, Wigby, Boone, Partridge, & Chapman, 2008; Jensen, McClure, Priest, & Hunt, 2015; Lee et al., 2008), which may result in them becoming more aggressive when competing over a protein‐rich food source (such as the yeast paste we used in this experiment). Mated females from both treatments laid more eggs than virgin females and this corresponded to an increase in contest duration in both treatments. However, high density females experienced a greater increase in contest duration after mating than low density females (compare the difference between HMHM vs. HVHV with LMLM vs. LVLV in Figure 4), despite laying fewer eggs than low density females (Figure 1b). This supports our previous conclusion that egg production and aggression are not obligatorily coupled, and instead, the post‐mating aggression response is more directly stimulated by transfer of sperm and other components of the male ejaculate (Bath et al., 2017). Note that the relatively low rate of egg production in this study is likely a result of females receiving no live yeast prior to fights; nonetheless, the egg numbers here are consistent with our previous work (Bath et al., 2017).

Male ejaculates stimulate a range of post‐mating responses in female *D. melanogaster*, ranging from increased egg production, reduced sexual receptivity and increased aggression (Avila, Sirot, LaFlamme, Rubinstein, & Wolfner, 2011; Bath et al., 2017; Kubli & Bopp, 2012). Female developmental environment can influence how much ejaculate males transfer to females, as well as how responsive females are to those ejaculates. Males transfer smaller ejaculates to smaller females in *D. melanogaster*, but not in direct proportion to the female's body weight (Lüpold et al., 2011; Wigby et al., 2016); that is, smaller females receive smaller *absolute* ejaculates, but these are still larger *relative* to body size than for larger females. High density females may therefore receive a larger relative dose of seminal fluid proteins—including aggression‐stimulating ejaculate components—than low‐density females, resulting in the observed greater increase in post‐mating aggression.

We also observed a developmental environment‐dependent effect on contest outcome—mated females raised at high density were more likely to initiate attacks and win than virgin females from the same density treatment, but mating status had no effect on contest outcome in low density females. This result could be due to increased relative ejaculate received by high density females or by an increased sensitivity to ejaculates in high density females. Additionally, the developmental environment could be a sign of the competition and harassment level likely to be encountered in adulthood, with high density females “predicting” a higher level of adult competition than low density females. Male *D. melanogaster* raised at high larval density had larger accessory glands than males raised at low larval density, which is associated with increased pre‐ and post‐reproductive success, and appears to be a reaction to perceived adult competition (Bretman, Fricke, Westmancoat, & Chapman, 2016). *Drosophila melanogaster* larvae that encounter crowded conditions experience higher rates of cannibalism and develop more teeth as a plastic response to this competition (Vijendravarma, Narasimha, & Kawecki, 2013). High density females may respond to an increased level of perceived adult competition by being more sensitive to male ejaculate proteins, and more likely to endure in contests for oviposition sites and food patches. High density females mate less frequently than low density females over a given period of time, but do not display a higher rate of egg production. This suggests that high density females may be more sensitive to remating suppression from ejaculate components, but are less sensitive or able to respond to ejaculate components stimulating egg laying (Morimoto et al., 2016).

Alternatively, females raised on high larval density may place a higher value on the contested resource (proteinaceous yeast paste). High density females may value food more as adults due to restricted access during development, which appears in this study as smaller adult body size and lower egg production. High density females spent longer feeding than low density females, suggesting a greater need for food. An increased perception of value may lead to an increased motivation to compete for the resource, resulting in high density females persisting for longer in contests than expected (Enquist & Leimar, 1987). The increased aggressive response we observed in high density mated females may be due to these females attempting to compensate for deprivation during development when egg production has been stimulated.

Our results potentially provide support for a “desperado” effect in female *D. melanogaster* (Grafen, 1987). The “desperado” effect describes a situation where individuals that should lose when following the regular rules governing a contest will still engage in competition, as they cannot gain any fitness by not engaging at all (Elias et al., 2010). If there is always a class of individuals that will lose contests if they obey the rules of the contest (i.e., that larger females will always win), and they cannot acquire reproductive success without competing, smaller females have little to lose from competing, even if their chances of winning are relatively low. High density females have a small chance of winning against low density females due to the large difference in body size, but, once high density females have mated, they need access to protein to lay their eggs, which they may be unable to access unless they engage with low density females. Theory predicts that smaller individuals may be more aggressive than larger individuals, even when they can accurately assess their lower chances of winning an encounter if body size does not always predict contest outcome (Morrell, Lindström, & Ruxton, 2005). Our results suggest that although low density females typically beat high density females, there was a greater increase in aggression after mating in high density females, accompanied by a slight improvement in how many encounters they won—suggesting high density mated females have increased motivation to challenge the traditional contest rules, and potentially improve their access to vital resources.

Although we found an increased likelihood of mated females winning against virgins amongst high larval‐density competitors, larval density itself was the strongest predictor of contest outcome in female *D. melanogaster*. There were large differences in body size arising from the two larval density treatments—low density females weighed almost twice as much as high density females, which is a significant barrier to overcome in a physical contest. If the density of larvae in the developmental environment is an indicator of adult competition or influences perceived value of resources as adults, and this determines success in adult competition, we would expect females from high larval density (small body size) to initiate—and perhaps win—more contests against large females from low larval density developmental environments. However, we observed the exact opposite, perhaps because the difference in body size and condition between the two treatments overrides any differences in developmental plasticity in aggressiveness. Body size plays a major role in determining physical contests in a large range of animals, where larger individuals are often more likely to win in direct encounters, resulting in more control of a patchy resource and higher reproductive success (Cowlishaw & Dunbar, 1991; Han & Jablonski, 2010; Kelly, 2008; McCann, 1981; McGraw et al., 2007; Moczek, 1998). Larger male *D. melanogaster* win more contests than smaller males and are able to consistently prevent smaller males from being present in their territory (Dow & von Schilcher, 1975; Hoffmann, 1987; Partridge & Farquhar, 1983). Our results suggest that females show a similar effect of body size, with low density females winning a higher proportion of encounters, particularly when facing smaller high density females. Females also appear to be able to detect differences in body size, and potentially differences in contest ability, as all females initiated more attacks when their competitor was from high larval density; that is, females started more contests when their opponent was smaller and so their chance of winning was higher.

## 5 CONCLUSION

We found a strong influence of larval density on adult female–female competition in *D. melanogaster,* and a strong interaction with mating status, whereby individuals raised at different larval densities demonstrated different strengths of response to mating in their competitive encounters with other females. The results from this experiment suggest that evaluating both developmental environment and aspects of reproduction could provide useful insights into understanding the dynamics and outcomes of female–female competition, and how this differs from, or is similar to, male–male competition. Expanding such research to include species with gestation, or prolonged maternal care, may help to increase our understanding of the overall lifetime influences on female aggression.

## AUTHORS’ CONTRIBUTIONS

E.B. and S.W. designed the experiment. E.B. and J.M. conducted the experiment. E.B. scored and analysed the behavioural data. E.B., J.M. and S.W. wrote the manuscript. All authors gave final approval for publication.

## DATA ACCESSIBILITY

Data are publicly available on the Oxford University Research Archive (https://doi.org/10.5287/Bodleian:E9Kz6RzEP; Bath, Morimoto, & Wigby, 2018).
