# Supplementary material for: The developmental environment modulates mating‐induced aggression and fighting success in adult female Drosophila
Source: Funct Ecol. 2018 Sep 28;32(11):2542–52. doi: 10.1111/1365-2435.13214 (PMC6472669; doi:10.1111/1365-2435.13214)
Supplement: Supplementary file 1 [file FEC-32-2542-s001.pdf]

Functional Ecology BRITISH ECOLOGICAL SOCIETY

Early-life competition determines how aggressive female fruit flies get over food

Eleanor Bath, Juliano Morimoto, Stuart Wigby

Competition over resources early in life can have dramatic effects on individuals' outcomes later in life. For example, how much and what type of food you eat when young can determine adult body size or have long term health implications, a finding which applies widely from fruit flies to humans. For animals, this early-life competition can also affect adult competition over resources such as food or mates. However, most of the work investigating how early-life competition influences adult competition has focused on males. Little is known about how juvenile and adult competition interact in females.

We tested how early-life competition influences female-female competition in the fruit fly. Fruit flies go through a larval stage, during which time they will compete with other larvae for food during development, occasionally even resorting to cannibalism if resources are scarce. Females will also compete with each other over food as adults. We experimentally altered larval density to change the amount of competition individuals faced. Females raised at high density had to compete more for food during development than females raised at low density, and as a result grew into smaller adults. When pairs of adult females fought over food, we found that low density females (who were larger adults) were much more likely to win fights than high density females.

In previous work, we showed that mating increases aggression in female fruit flies, so we also wondered whether the developmental environment could influence the relationship between mating and aggression – i.e. does a female's larval experience determine how much more aggressive mating makes her?

Interestingly, mating increased high density females' chances of winning, but had no effect on low density females. Mating

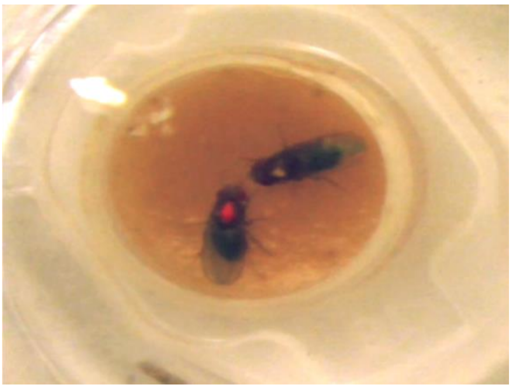

increased the amount of time low and high density females spent fighting over food, but this effect was particularly strong for high density females. Our results suggest that high density females are more sensitive to the aggression-stimulating effects of mating.

In summary, we found that early-life experience not only influenced how likely females were to succeed in adult competition, but also influenced how females respond to male ejaculates that stimulate aggression. Our results suggest that investigating the developmental environment and aspects of reproduction could provide interesting insights into understanding competition across a wide range of animals.
