# Supplementary material for: The developmental environment modulates mating‐induced aggression and fighting success in adult female Drosophila
Source: Funct Ecol. 2018 Sep 28;32(11):2542–52. doi: 10.1111/1365-2435.13214 (PMC6472669; doi:10.1111/1365-2435.13214)
Supplement: Supplementary file 2 [file FEC-32-2542-s002.pdf]

Appendix S1: Supplementary tables containing AIC evaluations of statistical models

Supplementary Table 1.a: Feeding duration model comparisons

Model	Terms included in the model	AIC	Deviance
Null	Intercept	6849.8	215.09
Best	FM + FD + CD	6837.6	206.59
2nd	FM + FD	6840.6	208.31
3rd	FM + FD + CD + CM	6837.7	206.59
4th	FM + FD + CD + FD*CD	6838.3	206.74
5th	FM + FD + CD + FM*CD	6838.4	206.76
6th	FM + FD + CD + FM*FD	6839.6	207.07
Full	All main effects and 2-, 3-, and 4-way interactions	6855.1	204.28

Legend: FM = Focal mating status, FD = focal larval density, CM = Competitor mating status, CD = Competitor larval density, * = interaction between factors.
The best model is highlighted in green, with models within 5 AICs highlighted in yellow, as they cannot be discounted as they are not significantly different from the best model.

Supplementary Table 1.b: Model estimates from best model for feeding duration

	Df	Estimate	SE	Deviance	P-value
FM	1	-0.1896	0.0474	4.3975	0.00003
FD	1	-0.1305	0.0482	2.3806	0.00200
CD	1	-0.1066	0.0482	1.2304	0.02628

Estimates and standard errors are reported on a log-scale as that was the transformation used in the model. They have not been back-transformed. P-value indicates whether the factor had a significant effect on feeding duration. Degrees of freedom are added sequentially. Significant values at $P < 0.05$ are highlighted in red. FM = Focal mating status, FD = focal larval density, CD = Competitor larval density

Supplementary Table 2.a: Contest initiations model comparison

Model	Terms included in the model	AIC	Deviance
Null	Intercept	1290.6	249.31
Best	CD + FD + FM + FM*FD	1236.8	249.52
1st	CD + FD + FM	1237.8	252.51
2nd	CD + FD + FM + CM + FM*FD	1237.9	248.59
3rd	CD + FD + FM + FM*FD + FD*CD	1238.2	248.85
4th	CD + FD + FM + FM*FD + FM*CD	1238.3	248.98
Full	All main effects and 2-, 3-, and 4-way interactions	1243.0	249.66

Legend: FM = Focal mating status, FD = focal larval density, CM = Competitor mating status, CD = Competitor larval density, * = interaction between factors.
The best model is highlighted in green, with models within 5 AICs highlighted in yellow, as they cannot be discounted as they are not significantly different from the best model.

Supplementary Table 2.b: Model estimates from best model for contest initiations

	Df	Estimate	SE	Deviance	P-value
CD	1	-0.7234	0.1196	23.1496	0.0000015
FD	1	0.4577	0.1623	23.1582	0.0000015
FM	1	-0.7472	0.1746	20.0707	0.0000075
FM * FD	1	0.4053	0.2337	2.9931	0.0836200

Estimates and standard errors are reported on a log-scale as that was the transformation used in the model. They have not been back-transformed. Degrees of freedom are added sequentially. P-value indicates whether the factor had a significant effect on contest initiations. Significant values at P < 0.05 are highlighted in red, and p-values below 0.1 are highlighted in yellow.
FM = Focal mating status, FD = focal larval density, CM = Competitor mating status, CD = Competitor larval density, * = interaction between factors.

Supplementary Table 3.a: Proportion of encounters won model comparison

Model	Terms included in the model	qAIC	Df
Null	Intercept	791.8	1
Best	FD + FM + CD + CM + FD*FM + CD*CM	610.4	7
1st	FD + CD	612.6	3
2nd	FD + CD + CM	612.9	4
3rd	FD + FM + CD	613.1	4
4th	FD + CD + FD*CD	614.6	4
5th	FD + FM + CD + CM	612.0	5
6th	FD + CD + CM + FD*CD	614.8	5
7th	FD + FM + CD + CM + FD*FM	611.8	6
8th	FD + FM + CD + CM + FM*CD	614.0	6
9th	FD + FM + CD + CM + FM*CM	614.0	6
10th	FD + FM + CD + CM + FD*CD	614.0	6
11th	FD + FM + CD + CM + FD*CM	613.8	6
12th	FD + FM + CD + CM + CD*CM	612.2	6
13th	FD + FM + CD + CM + FD*FM + FD*CM	613.0	7
14th	FD + FM + CD + CM + FD*FM + FM*CD	613.5	7
15th	FD + FM + CD + CM + FD*FM + FD*CD	613.8	7
16th	FD + FM + CD + CM + FD*FM + FM*CM	613.8	7
17th	FD + FM + CD + CM + FD*FM + FD*CM + CD*CM	612.3	8
18th	FD + FM + CD + CM + FD*FM + FM*CD + CD*CM	612.3	8
19th	FD + FM + CD + CM + FD*FM + FD*CD + CD*CM	612.4	8
20th	FD + FM + CD + CM + FD*FM + FM*CM + CD*CM	612.4	8
21st	FD + FM + CD + CM + FD*FM + FD*CM + FM*CM + FM*CD + CD*CM + FD*FM*CM + FM*CD*CM	614.4	12
Full	All main effects and 2-, 3-, and 4-way interactions	622.1	16

Legend: FM = Focal mating status, FD = focal larval density, CM = Competitor mating status, CD = Competitor larval density, * = interaction between factors.
The best model is highlighted in green, with models within 5 qAICs highlighted in yellow, as they cannot be discounted as they are not significantly different from the best model.

Supplementary Table 3.b: Model estimates for best proportion won model

	Df	Estimate	SE	Deviance	P-value
FD	1	1.3446	0.2106	539.71	< 2e-16
FM	1	-0.5921	0.2334	16.67	0.12701
CD	1	-1.3713	0.2118	789.08	< 2e-16
CM	1	0.5558	0.2327	20.42	0.09119
FD * FM	1	0.6390	0.3227	16.86	0.12476
CD * CM	1	-0.5731	0.3220	22.84	0.07405

Estimates and standard errors are reported on a log-scale as that was the transformation used in the model. They have not been back-transformed. Degrees of freedom are added sequentially. P-value indicates whether the factor had a significant effect on the proportion of encounters won. Significant values at $P < 0.05$ are highlighted in red, and p-values below 0.1 are highlighted in yellow. FM = Focal mating status, FD = focal larval density, CM = Competitor mating status, CD = Competitor larval density, * = interaction between factors.

Supplementary Table 4.a: Contest duration model comparison

Model	Terms included in the model	AIC	Deviance
Null	Intercept	1394.8	131.57
Best	FM + CM + CD + FM*CD	1369.5	114.66
1st	FM + CM + CD + FM*CD + FM*CM	1370.5	114.21
2nd	FM + CM + CD	1371.1	116.42
3rd	FM + FD + CM + CD + FM*CD	1371.3	114.57
4th	FM + CM + CD + FM*CD + CM*CD	1371.3	114.59
5th	FM + FD + CD + CM + FM*FD + FM*CD + FD*CD + FM*CM + FD*CM + CD*CM + FM*FD*CD + FM*CD*CM	1369.6	107.34
6th	FM + FD + CD + CM + FM*FD + FM*CD + FD*CD + FM*CM + CD*CM + FM*FD*CD + FM*CD*CM	1369.9	108.44
7th	FM + FD + CD + CM + FM*FD + FM*CD + FD*CD + FM*CM + FD*CM + CD*CM + FM*FD*CD	1370.0	108.51
8th	FM + FD + CD + CM + FM*FD + FM*CD + FD*CD + FM*CM + FD*CM + CD*CM + FM*FD*CD + FM*CD*CM + FD*CD*CM	1370.8	106.94
9th	FM + FD + CD + CM + FM*FD + FM*CD + FD*CD + FM*CM + FD*CM + CD*CM + FM*FD*CD + FM*CD*CM + FM*FD*CM	1371.3	107.19
10th	FM + FD + CD + CM + FM*FD + FM*CD + FD*CD + FM*CM + FD*CM + CD*CM + FM*CD*CM	1374.8	110.89
Full	All main effects and 2-, 3-, and 4-way interactions	1374.0	106.61

Legend: FM = Focal mating status, FD = focal larval density, CM = Competitor mating status, CD = Competitor larval density, * = interaction between factors.
The best model is highlighted in green, with models within 5 qAICs highlighted in yellow, as they cannot be discounted as they are not significantly different from the best model.

Supplementary Table 4.b: Model estimates for best contest duration model

	Df	Estimate	SE	Deviance	Pr(>Chi)
FM	1	-0.5145	0.13706	8.6977	0.00002
CM	1	-0.3074	0.09538	5.2184	0.00102
CD	1	-0.3330	0.13531	1.2380	0.10964
FM * CD	1	0.3587	0.18886	1.7624	0.05629

Estimates and standard errors are reported on a log-scale as that was the transformation used in the model. They have not been back-transformed. Degrees of freedom are added sequentially. P-value indicates whether the factor had a significant effect on contest duration. Significant values at $P < 0.05$ are highlighted in red, and p-values below 0.1 are highlighted in yellow. FM = Focal mating status, FD = focal larval density, CM = Competitor mating status, CD = Competitor larval density, * = interaction between factors.

Supplementary Table 4.c: Model estimates for full contest duration model

	Df	Estimate	SE	Deviance	P-value
FM	1	-0.81288	0.24138	8.6977	0.00003
FD	1	0.11217	0.29149	0.0348	0.79157
CD	1	-0.24991	0.24138	1.4302	0.09023
CM	1	0.11091	0.32517	5.0254	0.00150
FM * FD	1	0.52960	0.40730	0.7361	0.22428
FM * CD	1	0.68567	0.36798	1.5590	0.07698
FD * CD	1	-0.02589	0.38144	1.1201	0.13387
FM * CM	1	-0.20915	0.40630	0.7042	0.23462
FD * CM	1	-0.71575	0.46679	0.9430	0.16900
CD * CM	1	-0.49073	0.42065	0.0001	0.98973
FM * FD * CD	1	-1.10974	0.57469	2.8111	0.01756
FM * FD * CM	1	0.38164	0.59319	0.2800	0.45356
FM * CD * CM	1	0.62213	0.61443	1.0453	0.14760
FD * CD * CM	1	0.49093	0.58929	0.5751	0.28276
FD * FM * CD * CM	1	-0.08196	0.83948	0.0048	0.92219

Estimates and standard errors are reported on a log-scale as that was the transformation used in the model. They have not been back-transformed. Degrees of freedom are added sequentially. P-value indicates whether the factor had a significant effect on contest duration. Significant values at $P < 0.05$ are highlighted in red, and p-values below 0.1 are highlighted in yellow. FM = Focal mating status, FD = focal larval density, CM = Competitor mating status, CD = Competitor larval density, * = interaction between factors.
